# Bereaved informal carers’ experience of an interventional clinical research project at the end-of-life: a qualitative interview study

**DOI:** 10.1186/s12904-025-01872-6

**Published:** 2025-10-29

**Authors:** Miriam White, Michael Connolly, Andrew Davies

**Affiliations:** 1 Royal Surrey County Hospital, Guildford, UK; 2 University of Surrey, Guildford, UK; 3 University College Dublin, Our Lady’s Hospice & Care Services, Dublin, Ireland; 4 Trinity College Dublin, University College Dublin, Our Lady’s Hospice & Care Services, Dublin, Ireland

**Keywords:** Clinically assisted hydration, Palliative care, Terminal care, Informal carers, Interviews

## Abstract

**Background:**

This qualitative study was undertaken to obtain feedback from informal carers about their experiences of involvement in a cluster randomised trial of clinically-assisted hydration in the last days of life (“CHELsea II trial”).

**Methods:**

Informal carers that had taken part in the trial post-bereavement postal survey, and had expressed an interest in taking part in further research, were approached about this post-bereavement qualitative study. Interviews were conducted remotely, using a semi-structured interview schedule that asked about the impact of the research on patient / their end-of-life care, the impact of the research on the informal carer, and the informal carers views on research at the end-of-life. The interview transcripts were thematically analysed.

**Results:**

Fifteen informal carers took part in the study prior to thematic saturation. Invariably informal carers reported that there was no negative impact on the patient or themselves, and were positive about taking part in future end-of-life research (if the situation occurred). The analysis generated three themes: (a) Purpose, helping, and no disruption; (b) Preparing for what was to come; and (c) Timing of research at the end-of-life.

**Conclusions:**

This study confirms that research can be undertaken in patients at the end-of-life without negatively impacting the experience. Moreover, patients and their informal carers want to take part in such research, as it provides them with purpose during this time, and gives them the chance of helping future patients.

**Trial registration:**

ISRCT Registry (registry number - ISRCTN65858561) – registered 14/09/2021.

## Background

Research in palliative care is challenging, and especially interventional clinical research projects, and/or clinical research projects involving patients in the last days of life. However, there is an imperative to undertake such research to ensure that patients (and their informal carers) receive the best care possible [1]. The CHELsea II trial is a cluster randomised trial of clinically assisted hydration (CAH) in the last days of life [2]. The aim of the CHELsea II trial is to evaluate CAH in patients in the last days of life, i.e. efficacy, tolerability, and health economic impact.

The CHELsea II trial included an informal carers’ postal survey, and a related informal carers’ qualitative interview study. The aim of these sub-studies was to obtain feedback from informal carers (families/friends) about their experiences of the CHELsea II trial. The objectives of these sub-studies were to determine: (a) informal carers’ perception of the impact of the research on the patient/their end-of-life care; (b) informal carers’ perception of the impact of the research on themselves; and (c) informal carers’ views on research at the end-of-life generally. This paper reports on the informal carers’ qualitative interview study.

## Methods

The CHELsea II trial is sponsored by the University of Surrey, and funded by the National Institute for Health and Care Research (NIHR) in the UK (Health Technology Assessment Programme - NIHR131687). Ethical committee approval has been granted by the Brighton and Sussex Research Ethics Committee (REC) (main REC for the UK: reference – IRAS 313640), and by the Scotland A REC (REC for adults with incapacity in Scotland: reference – 22/SS/0053-IRAS-317637). The CHELsea II trial is listed on ISRCT Registry (ISRCTN65858561).

### Study participants

Participants were recruited from individuals that returned the informal carer postal survey, and responded that they “would *(you)* be interested in providing additional feedback about your experiences?” Initially, consecutive individuals were recruited to the study. However, as the study progressed, individuals were selected in order to provide a varied group (i.e. in terms of sex, patient relationship, patient intervention). Nevertheless, most of the participants in this study were White British, which reflected the ethnicity of the participants in the CHELsea II trial.

### Participant recruitment

The CHELsea II trial involved 91 sites across England, Scotland, and Wales (i.e. hospices with inpatient units, hospitals with specialist palliative care services/units). Applicable individuals were sent a pack containing a covering letter, a participant information leaflet, a consent form, a contact details form, and a prepaid envelope (to return the consent form and contact details form).

### Interview format

As a result of the widespread locations of the study sites the interviews were undertaken remotely using Microsoft Teams^®^ software (Microsoft Corporation, Redmond). At the start of the interview the researcher introduced themself, and reminded the participants about the nature/purpose of the study, that the interview was being audio recorded, and that they could pause or stop the interview at any point (and for any reason). The participants were then asked to reaffirm their consent to take part in the study. The interviews were semi-structured (see Appendix 1 for the interview schedule), were conducted by a single researcher (MW), and were audio recorded for subsequent verbatim transcription (by MW). No field notes were taken. Participants were allowed to have someone with them, but that person was not allowed to contribute/answer questions. The interviews lasted 15–30 min. MW received training/mentoring from MC, who is an experienced qualitative researcher.

### Interview analysis

The interview transcripts were thematically analysed using the six-step method developed by Braun and Clarke [3, 4]: (1) familiarising yourself with your data; (2) generating initial codes; (3) searching for themes; (4) reviewing themes; (5) defining and naming themes; and (6) producing the report. The analysis was conducted by two researchers (MW, MC), initially independently and subsequently collaboratively, to limit potential interviewer biases. MW and MC initially read the interview transcripts in order to become familiar with the data and identify key concepts. NVivo^®^ version 12 software (Lumivero, Colorado) was used to systematically manage the data. Separately MW and MC inductively coded the interview transcripts line by line, capturing key data to generate initial codes. MW and MC met to review codes and discuss alignment and disparity in initial codes. Once this review was complete, agreed codes were searched for categories, which were then organised into themes. MW and MC further refined the categories and themes in order to reach consensus and produce the final agreed themes. Recruitment continued until there was agreed thematic saturation. This article conforms to the consolidated criteria for reporting qualitative research (COREQ) checklist [5].

## Results

Fifteen informal carers were interviewed prior to the development of thematic saturation: no interviews were stopped, and no participants were withdrawn from the study. Table 1 shows the characteristics of the included individuals.

**Table 1:** Participant characteristics

CHARACTERISTIC	NUMBER OF INDIVIDUALS (*n* = 15)
Sex	
Female	10
Male	5
Ethnicity	
White British	12
White Irish	1
No data	2
Relationship to patient	
Spouse	8
Child	4
Sibling	3
Intervention patient received	
Standard end-of-life care	7
Standard end-of-life care and clinically-assisted hydration	8

Three themes were generated from the analysis: (1) Purpose, helping and no disruption; (2) Preparing for what was to come; and (3) Timing of research at the end-of-life. Importantly, all of the participants were strongly in favour of undertaking research at the end-of-life, and expressed gratitude to the research teams as well as the clinical teams involved in the patient’s end-of-life care.

### Purpose, helping and no disruption

The majority of participants mentioned that the clinical trial had given the patient (and themselves) purpose in terms of “doing something positive” during the patient’s last days of life.

*“We wanted to*,* em [pause]*,* take part in something that was positive in the middle of something that was just*,* absolutely tragic*,* really” (Participant A)*.

Indeed, all the participants stated that the patient’s involvement in the trial should help others in the future, and that they (patients and informal carers) were grateful for the chance to take part in the trial.

*“…you’re not gonna be here long. And it’s gonna help others. I think it’s absolutely brilliant” (Participant B)*.

*“ I think it was just like*,* well look*,* if it helps somebody else erm*,* then that’s good*,* that’s something positive that we can do in a really terrible situation*,* so*,* so that’s*,* that’s how I felt about it” (Participant C)*.

Moreover, several participants commented how proud they were that the patient had decided to take part in the trial even though they might not themselves directly benefit.

None of the participants felt that the trial had disrupted the end-of-life care that the patient received, or indeed had interfered with their (or others) interactions with the patient during this period.

*“…once I’d sign the consent form*,* I think that was on the first day that he was in the hospice possibly*,* um*,* I literally didn’t think about it again at all…” (Participant D)*.

Notably, all of the participants commented on the excellence of the end-of-life care that the patient received, with three participants suggesting that the patient had received additional attention as a result of the regular/4 hourly observations undertaken during the trial.

*“I think they looked after him even more” (Participant E)*.

### Preparing for what is to come

Several of the participants noted that they were unprepared for the death of the patient, and especially unsure about the process of dying. However, trial-related discussions (pre-consent, during trial) helped to address pertinent questions.

*“I think it’s obviously something*,* that we weren’t prepared for*,* - understanding how the general end-of-life goes” (Participant F)*.

Two participants expressed surprise that there was a lack of evidence about the use of fluids at the end-of-life (and so the need for trial).

*“Why is it taking till now before you’re going for this relatively basic information? This is - you know - this is - it shouldn’t - this should be known already” (Participant G)*.

*“I just thought it was a bit bizarre that - you know - that we have to figure out how much fluid someone needs in this sort of day and age” (Participant F)*.

One participant commented on the use of concomitant “sedative” patient medication, which reduced their opportunity to fully communicate with each other. Nevertheless, this participant was overall satisfied with the end-of-life care that the patient had received.

*“I supposed from a selfish point of view*,* [pause] I would have preferred*,* for my wife to stay conscious for a bit longer*,* but also I didn’t*,* didn’t want her to suffer at all” (Participant H)*.

### Timing of research at the end-of-life

The majority of the participants had provided assent for the patient to take part in the trial, or for the patient to remain in the trial after a loss of mental capacity. Participants were happy to have been asked, and especially when they were aware of the patient’s opinions about the trial (and about research generally). However, participants thought it was important that the whole family was involved in the decision, rather than just themselves as the selected “personal consultee” [2].

“*I think I was happier that I was being asked to sign things because I think that’s where the anxiety came*,* because I was like*,* well I don’t think my mum’s in any sort of place to actually start signing things” (Participant C)*.

*“I did have to speak to his children first*,* um and get their agreement because I didn’t just want to say yes*,* I wanted them to be happy” (Participant E)*.

One participant mentioned that the patient had been made aware of the trial before they became eligible for the trial, and that discussing their views at that time had made the decision much easier when the moment arrived. Relatedly, two participants expressed a desire that something similar would have occurred with their family member.

*“I thought it was odd that you didn’t ask her [pause]… because*,* er*,* [pause] if you’d asked a day*,* a couple of days before*,* she would have been able to answer herself” (Participant G)*.

Participants were invariably complimentary about the way the trial was conducted, and the attitude and conduct of the research team: in terms of the consent process, they felt they were given sufficient information (and that their questions answered), and that there was no pressure to take part, or to make a quick decision.

*“We*,* I spent a lot of time at the hospital*,* and you never felt so you’re being encroached on*,* so the approach by the*,* the actual medics and nurses were*,* was superb really” (Participant I)*.

One participant commented that some nurses appeared unsupportive of the trial, and “disgruntled” at having to give the fluids via gravity (and not via an infusion pump). However, the participant was content for the patient to remain in the study, and to continue with the fluids.

*“…so there were a lot of technical issues that seemed to um bother the staff*,* which then obviously bothered us too and*,* um there were some people*,* some nurses who were actually against the study*,* so that also had a slight impact” (Participant B)*.

## Discussion

This qualitative study is somewhat unique in terms of seeking the opinions of informal carers of patients involved in an interventional randomised clinical trial at the end-of-life [6, 7]. Indeed, we only know of one analogous publication [7]. Importantly, these informal carers were active contributors to the trial in terms of either providing assent for the patient to take part in the trial, or providing assent for the patient to remain in the trial when the patient lost mental capacity (close to death) [2]. It should be noted that assent from informal carers was the main method of obtaining agreement to participate in the CHELsea II trial, and whilst a single person acted the “personal consultee”, there was often a wider discussion within the close family. In some (minority) cases, the patients were aware of the trial before they became eligible, and so the personal consultee’s decision was guided by the patient’s formerly expressed thoughts.

Unsurprisingly, the findings of this qualitative study mirror those of the related postal survey. However, the qualitative study generated additional information, which should inform the design of future studies in this cohort of patients. The primary motivation for taking part in the trial was to help future patients, and this was important for both the patients and their informal carers. Other researchers have reported similar findings [7, 8]and also that informal carers are proud of patients for participating in end-of-life research [7]. Uniquely, this study highlights that the trial gave the patients and their informal carers a specific purpose during those last days of life.

The study intentionally included a range of participants in terms of age, sex, and relationship to the patient: the participants were mostly white British, which reflects the predominant ethnicity of the patients recruited to the CHELsea II trial (and the patients referred to specialist palliative care services within the United Kingdom). Nonetheless, the latter limits the generalisability of the study results. Previous studies suggest that persons who did not volunteer for similar interviews may be different from those that did volunteer for these interviews [9]. Nevertheless, the findings of this qualitative study mirror the findings of the related postal survey, which suggests that the interview sample was representative of the wider group.

Some of the participants became visibly distressed during the interview, but none of them asked for the interview to be paused or stopped. Furthermore, although explicitly suggested (by the interviewer), none of them asked for further follow up for their distress, e.g. referral to general practitioner, referral for grief support. Other studies have reported similar happenings, although informal carers tend to report that taking part in these types of interviews are generally beneficial [10, 11]. Importantly, the distress was not related to the trial, or to the end-of-life care (but to the death of the patient). Indeed, none of the informal carers felt that the trial had disrupted the patients’ end-of-life care, and three of the informal carers felt that they had received better end-of-life care as a result of participation. Other researchers have reported similar findings [7].

## Conclusions

This study confirms that research can be undertaken in patients at the end-of-life without negatively impacting the experience. Moreover, patients and their informal carers want to take part in such research, as it provides them with purpose during this time, and gives them the opportunity to help future patients.

## Appendix

### Interview schedule

1. How did you feel when it was suggested that your relative/friend take part in the study?
2. Were you asked to sign a consent form agreeing for your relative/friend to take part in the study?
3. If YES, how did you feel about being asked to sign the consent form?
  If NO (and the patient consented), how did you feel about your relative/friend agreeing to take part in the study?
  If NO (and a nominated consultee consented), how did you feel about a healthcare professional deciding that your relative/friend could take part in the study?
4. How do you feel now about your relative/friend taking part in the study?
5. Do you think that the study affected the care received by your relative/friend?
6. Do you have any other comments about the study (positive, negative)?
7. What are your general thoughts about doing research in people at the end-of-life?

## Data Availability

No datasets were generated or analysed during the current study.

## Abbreviations

CAH: clinically-assisted hydration
HTA: Health Technology Assessment
IRAS: Integrated Research Application System
NIHR: National Institute for Health and Care Research
REC: Research Ethics Committee
